# Sympathetic Duet: Simultaneous Stellate Ganglion Block and Thoracic Epidural Analgesia in the Successful Termination of Ventricular Storm

**DOI:** 10.7759/cureus.59867

**Published:** 2024-05-08

**Authors:** Garret Heiberger, Clayton Busch, Steven Havlik, Antonius Gunawan, Timothy Woodin, Richard Bryant

**Affiliations:** 1 Anesthesiology, Kansas City University of Medicine and Biosciences, Kansas City, USA; 2 Anesthesiology, The Ohio State University Wexner Medical Center, Columbus, USA

**Keywords:** refractory ventricular fibrillation, refractory ventricular tachycardia, ventricular storm, stellate ganglion block, thoracic epidural analgesia

## Abstract

This article discusses the management of ventricular storm (VS), a condition characterized by recurrent episodes of sustained ventricular tachycardia or fibrillation, which poses a significant risk of mortality. Prompt intervention is crucial, yet surgical options are often limited due to the patient's unstable condition. This case report presents a 47-year-old female who experienced VS during a planned surgical procedure. Despite initial stabilization, she continued to experience life-threatening arrhythmias, prompting the implementation of simultaneous stellate ganglion block (SGB) and thoracic epidural analgesia (TEA) catheters. This combined approach successfully controlled the arrhythmias, allowing for subsequent surgical interventions. The article emphasizes the potential of SGB and TEA as a bridge to definitive therapies for refractory VS, highlighting the need for further research to solidify their role in clinical practice.

## Introduction

Ventricular storm (VS) is defined as three or more episodes of sustained ventricular tachycardia (VT) or ventricular fibrillation (VF) in 24 hours. It is associated with high mortality rates, exceeding a 17-fold increase within the first three months compared to patients without VS [1]. Episodes of VS result in over 400,000 deaths per year in the U.S., and its presence confers an eightfold increase in the risk of death even when an implantable cardioverter-defibrillator (ICD) is present [2].

Prompt intervention is crucial to control VS and prevent cardiac arrest. Initial measures include both pharmacologic and non-pharmacologic measures, including electrolyte optimization, antiarrhythmic medications, and sedation. Surgical interventions such as sympathectomy and heart transplants may be considered when medical management alone has failed. Unfortunately, unstable ventricular arrhythmias preclude these patients as surgical candidates. This is where temporary, chemical sympathectomies, such as a stellate ganglion block or thoracic epidural, may be utilized. The interruption of cardiac sympathetic tone helps to reduce episodes of ventricular arrhythmia [3,4]. These interventions may open a window that allows for surgical intervention or other means to resolve underlying cardiac pathology.

Prior studies and case reports have described sphenopalatine ganglion block (SGB) and thoracic epidural analgesia (TEA) utilized separately, both with and without catheters. Notably, stellate ganglion catheters have been found to have impressive cessation rates, with 90% defined as the absence of recurrence within 24 hours of the catheter [5]. When thoracic epidural catheters are used, 45% of patients saw at least an 80% reduction in the incidence of their incessant VT [4].

Prior studies have evaluated SGB and TEA both with and without catheters. However, in this case report, we describe simultaneous SGB and TEA continuous ropivacaine infusions, which successfully bridged a high VT/VF burden patient to definitive surgical therapies. No recurrence of VT was noted until six days after the initiation of these therapies. It was only after the catheters were removed that VT recurred.

## Case presentation

We present the case of a 47-year-old female transferred to a quaternary care center following an episode of VF arrest prior to an elective outpatient operation. The patient’s past medical history was significant for failed sudden cardiac death requiring subcutaneous implantable cardioverter-defibrillator (SCD) placement, premature ventricular contraction (PVC) status post-radio frequency ablation, obstructive sleep apnea on CPAP, hypothyroidism, and chronic back pain with L5-S1 disc herniation. The patient presented for a planned posterior lumbar L5-S1 discectomy at an outside hospital. A magnet was then applied to her automatic implantable cardioverter defibrillator (AICD) to disable it during surgery. After standard induction of anesthesia with propofol, lidocaine, rocuronium, and fentanyl, the patient was intubated and placed in a prone position for facilitation of surgical access. Shortly after transitioning to the prone position, the patient was noted to have a six-beat run of ventricular tachycardia, which evolved into ventricular fibrillation and cardiac arrest. Standard advanced cardiac life support (ACLS) protocols were initiated, and return of spontaneous circulation (ROSC) was ultimately achieved after one round of chest compressions, 1 mg of intravenous epinephrine, and a single defibrillation with 200 J. The patient then remained in sinus rhythm with frequent PVCs. The planned surgical case was canceled, and the patient was then transferred to the intensive care unit (ICU) intubated and sedated with continuous propofol infusion. 

The patient was transferred to a quaternary care center on post-operative day (POD) zero for evaluation by cardiology intubated, on a continuous propofol infusion, hemodynamically stable, and in normal sinus rhythm. Given hemodynamic and cardiovascular stability upon arrival, the decision was made to wean ventilatory support and liberate the patient from mechanical ventilation. 

**Figure 1:**
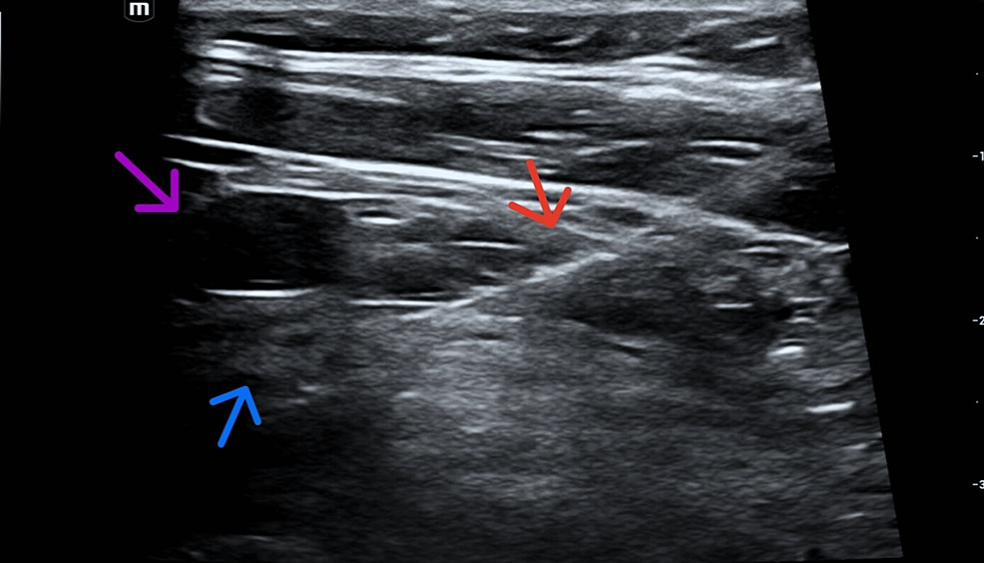
Ultrasound still image of stellate ganglion catheter placement. Red arrow: Tuohy needle and catheter; blue arrow: stellate ganglion; purple arrow: carotid artery.

After an uneventful night, a code blue was called on POD1 after the patient was found to be in polymorphic ventricular tachycardia. ROSC was achieved following the firing of the subcutaneous AICD. The patient was then started on IV amiodarone, and electrophysiology was consulted for further evaluation. Approximately 30 minutes following the firing of her AICD, the patient’s cardiac rhythm returned to polymorphic ventricular tachycardia/ventricular fibrillation and a subsequent Code Blue was called. The patient’s AICD continued to fire appropriately, initially terminating her VF with a return of sinus rhythm, however, her cardiac rhythm continued to devolve into repeat VF resulting in consistent firing of AICD. The patient was re-intubated and sedated with propofol to achieve Richmond Agitation-Sedation Scale (RASS) goal −4 to minimize sympathetic stimulation. The patient had endured 40 rounds of ACLS by this juncture. Cardiac surgery was consulted, and a bilateral sympathectomy was recommended. In a concurrent effort to provide analgesia for broken ribs sustained during ACLS and minimize sympathetic stimulation to the heart, the acute pain service was consulted, and thoracic epidural and left-sided continuous stellate ganglion catheters were placed on the afternoon of POD1 (Figure 1). The patient was initiated on continuous 0.2% ropivacaine infusion at 4 mL/h through the stellate ganglion catheter after a 20 cc bolus of the same solution. Additionally, a thoracic epidural catheter was placed at T5-T6 using 0.2% ropivacaine at 4 mL/h without a bolus. 

Following placement of a stellate ganglion catheter and thoracic epidural, the patient remained heavily sedated with RASS −4 on a propofol infusion with concurrent lidocaine and amiodarone infusions until definitive surgical intervention could be arranged. She remained without further episodes of malignant arrhythmia during this period. On POD4, the patient underwent bilateral sympathectomy to further minimize sympathetic drive to the heart. The left-sided stellate ganglion catheter was removed during the sympathectomy to facilitate the surgical approach. Given the lack of malignant arrhythmia and hemodynamic stability for 48 hours following her sympathectomy, the decision was made to remove the thoracic epidural catheter on post-sympathectomy day two.

On post-sympathectomy day three, the day after the removal of the thoracic epidural, the VT storm returned, including another code blue. The patient underwent serial shocks from her AICD but re-entered ventricular fibrillation shortly after each shock. Her malignant arrhythmia was refractory to IV metoprolol boluses and the subsequent initiation of a continuous esmolol infusion, in addition to previously started amiodarone and lidocaine infusions. Given refractory ventricular fibrillation, the decision was ultimately made to transfer the patient to the operating room with cardiac surgery for cannulation and the initiation of extracorporeal membrane oxygenation therapy as a bridge to definitive therapy with cardiac transplantation.

## Discussion

**Figure 2:**
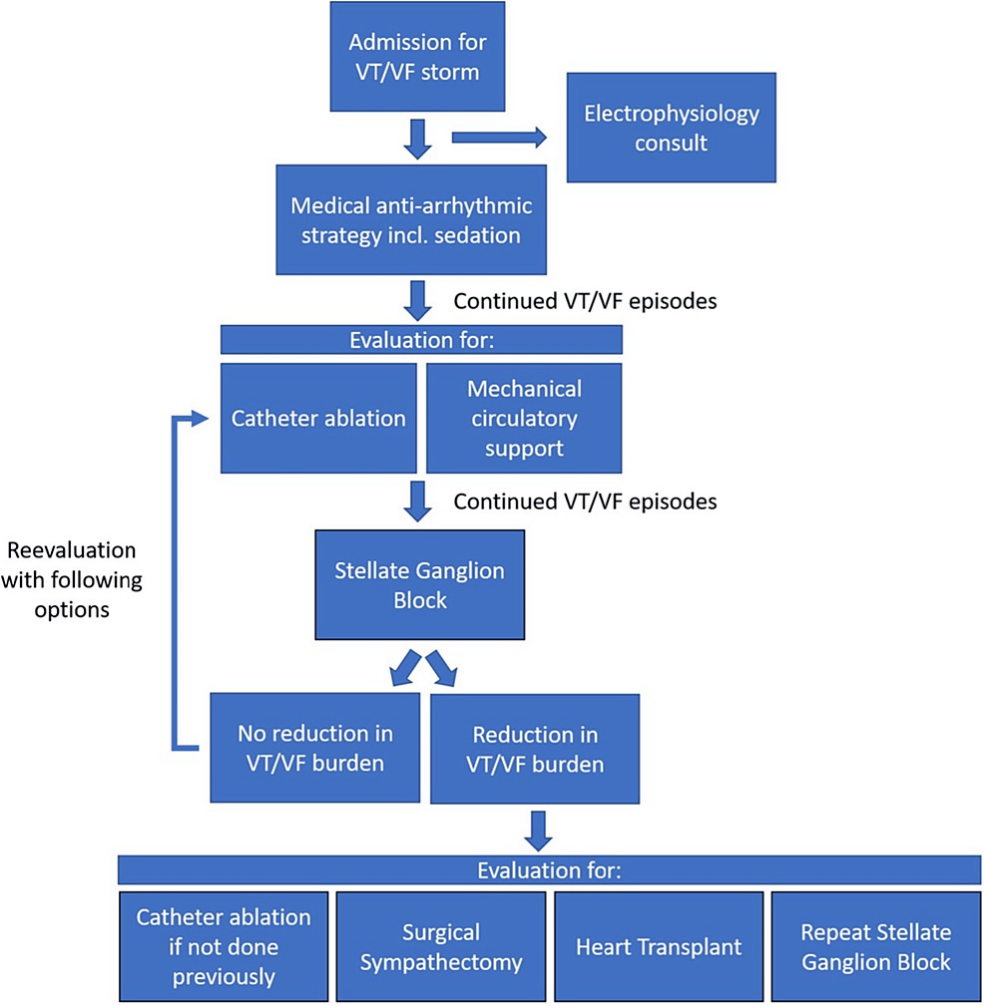
Modified Duke treatment algorithm for refractory ventricular arrhythmias. Used from Ganesh et al. with permission [7]. VT: ventricular tachycardia, VF: ventricular fibrillation.

The autonomic nervous system is a therapeutic target for VT as it plays a crucial role in the genesis of ventricular arrhythmias [6]. Treatment of VS includes antiarrhythmic drugs, addressing the reversible causes (electrolytes and ischemia), device-related therapies, and catheter ablations [3]. Surgical interventions should be considered in the event of failed medical management (Figure 2). This creates a catch-22 where a patient’s indication for a procedure is precisely what makes them an acutely poor surgical candidate. Sympathetic tone becomes a desirable target in the pre-surgical armamentarium. Sympathetic tone can be reduced in multiple locations throughout the sympathetic pathway. Reducing sympathetic tone centrally may be achieved through sedation. Peripheral targets include SGB and TEA via either a single dose injection or a continuous infusion via catheter placement. In this case report, the latter was implemented for both a SGB and a TEA. 

The stellate ganglion is the meeting point of pre- and post-ganglionic sympathetic nerves. This consolation of neuronal cell bodies is located anteriorly to the C7 or T1 vertebral body [8]. A left SGB is preferred, as there is evidence that it provides a more sympathetic tone to the heart compared to the right, particularly the ventricles [7,9]. Supporting this, Tian et al. reported no difference between bilateral and left SGB in a sample of 30 patients [10]. Ultrasound is the imaging modality of choice, as it is readily available, easy to use, and allows visualization of important structures, such as the carotids and vertebral arteries. Fluoroscopy, landmark techniques, and CT-guided techniques are also possible [7]. Ropivacaine injection is preferred, as it is less cardiotoxic than the more commonly used bupivacaine. Horner’s syndrome, perfusion index (PI), and increased temperature have been used to confirm a successful block, although they are neither sensitive nor specific. SGB is generally regarded as a safe procedure, although it is imperative to be aware of the feared complication of local anesthetic toxicity. Other complications include temporary hoarseness, hematoma, light-headedness, hypertension, and a brachial plexus block [11]. SGB as a treatment for VS has been supported by a meta-analysis of case reports as well as in multiple single-center case series [3,10,12]. Ganesh et al. notes a lack of prospective randomized clinical data comparing SGB to a standard of care and calls for a multicenter randomized trial to reliably cement the use of SGB for the treatment of VS [7]. Interestingly, and contrary to logical thought, SGB control of VS does not predict the success of sympathectomy, as seen in this case. Adequate control of VS was achieved through sympathetic suppression via SGB and TEA, yet ventricular arrhythmias persisted post-bilateral sympathectomy. Ganesh et al. suggests this may be explained by sympathetic tone being an amplifier, rather than a direct cause [7]. Findings in this case report are supportive of both statements.

TEA, more commonly used for its analgesic properties via interruption of nociceptive fibers, also decreases sympathetic cardiac input by interrupting segments C8-T4 proximal to the stellate ganglion. In this case report, it was useful for both modalities as the patient suffered multiple broken ribs secondary to her repeated cycles of ACLS. TEA is inherently a bilateral blockade, as a catheter is inserted into the thoracic epidural space and local anesthetics such as ropivacaine may be used. Successful treatment of refractory ventricular arrhythmias has been seen in a few cases [4]. The advantages of TEA include familiarity, as most anesthesiologists are comfortable performing such a procedure at the bedside without image guidance. Other considerations for TEAs include anticoagulation status, common in patients with VS, which increases the risk for complications with TEA, such as hematomas [7]. Further, markers of a successful TEA are lacking, which poses challenges in titration [9]. 

## Conclusions

Ventricular storm is associated with a high mortality rate and is often resistant to medical intervention. SGB and TEA have shown promise as a bridge to more permanent therapies. Where should a SGB fall in the treatment of VS has been asked previously, in addition, should the TEA be considered alongside SGB? This case report highlights the use of simultaneous SGB and TEA catheters in treating refractory VS, as a bridge to the more permanent therapies, sympathectomy and eventually cardiac transplant.
